# Predictors of Rehabilitation Referral Among Cardiovascular Surgical Patients

**DOI:** 10.3389/fcvm.2022.848610

**Published:** 2022-05-03

**Authors:** Laura Gómez González, Marta Supervia, José R. Medina-Inojosa, Joshua R. Smith, M. Esther López Blanco, M. Teresa Miranda Vivas, Francisco López-Jiménez, M. Olga Arroyo-Riaño

**Affiliations:** ^1^Department of Physical Medicine and Rehabilitation, Gregorio Marañón General University Hospital, Gregorio Marañón Health Research Institute, Madrid, Spain; ^2^Department of Cardiovascular Medicine, Mayo Clinic, Rochester, MN, United States; ^3^Division of Epidemiology, Department of Quantitative Health Sciences, Mayo Clinic, Rochester, MN, United States; ^4^Amita St. Joseph Hospital Internal Medicine Residency Program, University of Illinois Chicago, Chicago, IL, United States

**Keywords:** cardiovascular rehabilitation, cardiovascular disease, cardiac surgical procedures, secondary prevention, referral and consultation

## Abstract

**Objective:**

Cardiovascular disease (CVD) continues to be the leading cause of mortality globally. Cardiac rehabilitation (CR) programs act by modifying the evolution of CVD and mortality; however, CR programs are under-used. The aim was to determine the profile of patients that received rehabilitation after cardiac surgery.

**Patients and Methods:**

A retrospective observational study was conducted from January 2017 to December 2017 at a single center. The study sample was chosen among patients admitted to the Intensive Care Unit of the Hospital Gregorio Marañón/Gregorio Marañón General University Hospital. Socio-demographic and clinical variables were collected.

**Results:**

In the present study, 336 patients underwent cardiac surgery of which 63.8% were men and 87.1% had ≥1 cardiovascular risk factors. Of the total cohort, 24.7% were operated for ischemic heart disease, 47.9% valvulopathy, 11% underwent combined surgery, 3.6% cardiac transplantation, 6.5% aneurysms, and 3.9% congenital disease. In-hospital respiratory rehabilitation was prescribed to all patients. Only 4.8% of the patients received motor rehabilitation and 13.8% were referred to CR. We found higher referral rates among patients with more cardiovascular risk factors, <65 years of age, and those undergoing coronary surgery and heart transplantation. Age, ischemic heart disease, and overweight were independent predictors of CR referral.

**Conclusion:**

The benefit of CR programs after cardiac surgery is widely described; however, the referral rate to CR remains low. It is crucial to optimize referral protocols for these patients.

## Introduction

According to the latest report of the National Institute of Statistics regarding the causes of death in Spain in 2017, cardiovascular diseases (CVDs) continue to be the most prominent cause of death, representing 28.8% of total deaths (1). Further, ischemic heart disease is the leading cause of premature death in Spain. According to the Cardiovascular Surgery Registry in Spain (2), about 35,576 procedures are performed per year, of which 22,201 are major surgeries (3). Our hospital is one of the points of reference for this type of surgery, including heart transplantation.

Cardiac rehabilitation (CR) programs act by modifying the evolution of disease, controlling cardiovascular risk factors, and reducing morbidity and mortality (4). As a result, CR programs are currently considered as a Class IA indication in the treatment of heart disease (5). In clinical practice guidelines, CR is recommended for patients with CVD (6), heart failure (7), undergoing revascularization (8), and stable coronary heart disease (9). Similarly, these CR programs are indicated after heart valve disease (HVD) (10), heart transplant (HT) (11), and atrial fibrillation (12). Importantly, CR programs added to respiratory rehabilitation during and after admission, are cost-effective in these patients (13). To this point, in patients undergoing cardiac surgery, CR and respiratory rehabilitation during admission favor a faster recovery, reduce postoperative complications, and shorten hospital stay (14). Despite this, the implementation of CR programs is extremely low in many countries, including ours, with less than 10% of patients being rehabilitated (15). Therefore, it is critical to identify characteristics that predict CR referral in these surgical patients so that targeted interventions can be developed to improve CR referral.

There are reviews in the literature that suggest that CR programs are not available in more than 60% of countries around the world (16). In a recent study that analyzed the characteristics of different CR programs around the world, it was concluded that only 54.7% of the countries offered some type of CR program (17).

The objective of our study was to determine the socio-demographic factors and percentage of patients undergoing cardiac surgery, who performed an in-hospital respiratory rehabilitation program and outpatient CR program in order to identify patient characteristics that predict referral to CR programs.

## Materials and Methods

This retrospective observational study was approved by the Research Ethics Committee of the Gregorio Marañón Health Research Institute on July 2, 2018. The inclusion criteria for this study included: admission to the Intensive Care Unit of the Hospital Gregorio Marañón/Gregorio Marañón General University Hospital from January 2017 to December 2017 after cardiac surgery, not deceased during the post-operative period, not suffering from significant cognitive or severe physical impairment that hinders the rehabilitative treatment, having been assessed by the rehabilitation service during their admission, and living in Madrid.

In our hospital, the program starts from admission, beginning in the immediate postoperative phase (phase I) through supervised teaching of respiratory physiotherapy exercises, postural recommendations, mobility exercises, and stimulation of activities in a progressive way. After hospital discharge, the patient is referred as a candidate for the outpatient CR program (phase II), which consists of a supervised program of aerobic and strength exercises. It also includes psychological care and education for the control of cardiovascular risk factors and medication management. In this program, the objectives are improving functional capacity and offer the necessary knowledge for the safe practice of exercise and healthy lifestyle once the program is finished, continuing at home (phase III).

Information was collected from the electronic medical records, including socio-demographic data such as sex, age, and reference hospital as well as clinical data including cardiovascular risk factors, personal history of diseases (heart, respiratory, neurological, musculoskeletal, psychiatric diseases, or others), and the cause of surgery (ischemic heart disease, valve heart disease, heart transplant, aneurysm, congenital heart disease, and others). A body mass index greater than 25 was considered overweight.

In addition, the date of admission and hospital discharge, the date of surgery and whether there were post-surgical complications was collected. Information on the rehabilitation performed was collected that included respiratory, motor, and/or CR. Among those referred to CR, the date of CR referral, the referring department (i.e., cardiology or rehabilitation), the follow-up phase in which they were referred (i.e., during admission or in outpatient consultations), enrollment status into the CR program, and the start date of the program was also collected. It is important to note that regarding the referral to the CR program, we refer to the phase II of CR, performed outside the hospital in a center that belongs to the same health complex (outpatient CR program). Being a retrospective study, the data were collected months after cardiac surgery, and after the period for carrying out the CR program had elapsed, which allowed the data listed above to be collected. All data were collected manually through electronic medical records. As it was an observational study, the ethics committee of our center accepted the exemption of informed consent.

This data was stored on an Excel data collection sheet (Microsoft Office for Windows XP) and then statistical analysis was performed using IBM SPSS Statistics for Windows, Version 21.0. The results of the numerical variables were presented using their mean and standard deviation. For categorical variables, the results were shown in frequencies and percentages. The numerical variables with non-normal distribution were presented by means of their median and interquartile range (25th percentile; 75th percentile). The normality analysis was assessed with the Kolmogorov–Smirnov test. To compare differences between two or more groups, non-parametric tests were applied. The association between variables was analyzed using the Pearson Chi-square test. Regression models were created to evaluate the association between patient characteristics and CR referral, univariate modeling was performed adjusting for age and sex, and predictors that were significant were included for final multivariate modeling. Findings are presented as odds ratios (ORs) and 95% confidence intervals (CIs). Model assumptions were assessed graphically, and missing data were omitted. In all cases, two-tailed *p*-values < 0.05 were considered statistically significant.

## Results

**FIGURE 1:**
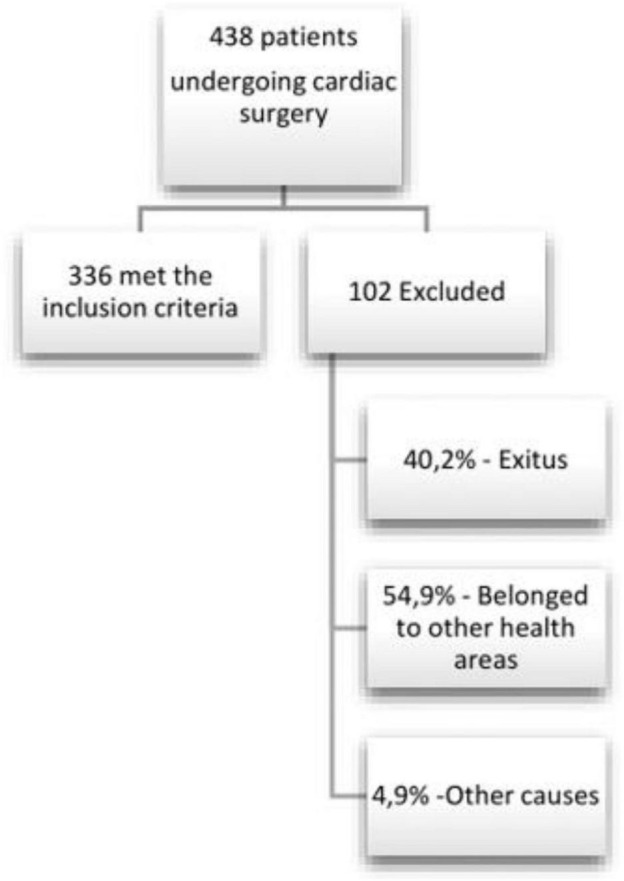
Flow chart of study design.

As shown in Figure 1, 438 patients underwent cardiac surgery in 2017 in our hospital. Of these patients, 102 patients were excluded: 40.2% (*N* = 41) due to exits, 54.9% (*N* = 56) for belonging to other health areas, and 4.9% (*N* = 5) for other causes (e.g., not assessed by the rehabilitating physician). There were 336 patients that met the inclusion criteria in the study. Of the patients included, 63.8% (*N* = 213) of these patients were men. The average age of the cohort was 64.16 years (SD = ±12.96) and 87.1% (*N* = 291) had at least one cardiovascular risk factor. The most frequent cardiovascular risk factors were hypertension (66.8%, *N* = 223), dyslipidemia (57.1%, *N* = 190), and smoking (36.9%, *N* = 122). Additionally, 29.1% (*N* = 97) suffered from diabetes mellitus, 10.6% (*N* = 35) were overweight or obese, and 0.6% (*N* = 2) consumed alcohol abusively. Only one of them used other illicit drugs (Table 1).

**TABLE 1:** Sociodemographic and clinical data of the total sample.

Variable	
Sex female (%)	36.2% (*N* = 121)
Age (%)	Average 64.16 (SD 12.96)
<65	48.5 (*N* = 162)
≥65	51.5 (*N* = 174)
Healthcare area (% of patients)	–
Our hospital	28.2 (*N* = 94)
Hospital from the area	54.4 (*N* = 181)
Hospital from a different area	17.1 (*N* = 57)
CVRF (%)	87.1
HTA	66.8 (*N* = 223)
DL	57.1 (*N* = 190)
DM	29.1 (*N* = 97)
Overweight/obesity	10.6 (*N* = 35)
Tobacco	36.9 (*N* = 122)
Alcohol	0.6 (*N* = 2)
Other drugs	0.3 (*N* = 1)
Previous CV comorbidities (%)	72.5
HF	19.8 (*N* = 66)
CHD	1.8 (*N* = 66)
IHD	22.8 (*N* = 76)
Myocardiopathy	5.1 (*N* = 17)
Pacemarker/ICD	5.4 (*N* = 18)
HVD	46.8 (*N* = 156)
Arrhythmia	29 (*N* = 97)
Other comorbidities (%)	78.4
Pulmonary disease	18.3 (*N* = 61)
Neurological disease	19.5 (*N* = 65)
Musculoskeletal disease	17.8 (*N* = 59)
Psychiatric disease	7.2 (*N* = 24)
Other disease	68 (*N* = 227)
Diagnosis to surgery (%)	–
IHD	24.7 (*N* = 83)
HVD	47.9 (*N* = 161)
IHD + HVD	11 (*N* = 37)
HT	3.6 (*N* = 12)
Aneurysms	6.5 (*N* = 22)
CHD	3.9 (*N* = 13)
Others	10.4 (*N* = 35)
Complications after surgery (%)	53.3 (*N* = 180)
Performed RR (%)	100 (*N* = 336)
Performed MR (%)	4.8 (*N* = 16)
Contraindications for CRP (%)	3 (*N* = 10)
Hospital length stay (days)	Average 23.34 (min 2, max 235)
Referred to CRP (%)	13 (*N* = 46)
Referred by ambulatory cardiology	10.2
Referred by ambulatory rehabilitation	4
Referred by hospital cardiology	5
Referred by hospital rehabilitation	3
Started CRP (%)	12.2 (*N* = 41)
Waiting days to start the program	Average 136.1 (SD 98.03, max 473, min 9)

*CVRF, cardiovascular risk factors; HTA, hypertension; DL, dyslipidemia; DM, diabetes mellitus; CV, cardiovascular; HF, heart failure; CHD, congenital heart disease; IHD, ischemic heart disease; ICD, implantable cardioverter defibrillator; HVD, heart valve disease; HT, heart transplant; RR, respiratory rehabilitation; MR, motor rehabilitation; CRP, cardiac rehabilitation program.*

Approximately 72.5% (*N* = 242) of the patients had suffered CVD before the surgical intervention. Of these, 19.8% (*N* = 66) had suffered heart failure, 1.8% (*N* = 6) congenital heart disease, 22.8% (*N* = 76) ischemic heart disease, 5.1% (*N* = 17) any cardiomyopathy, 5.4% (*N* = 18) had a pacemaker or an implantable automatic defibrillator, 46.8% (*N* = 156) heart valve disease, and 29% (*N* = 97) by some arrhythmia (e.g., AF, extra-systoles). Furthermore, 78.4% (*N* = 262) had suffered another type of CVD not mentioned above (Table 1).

**FIGURE 2:**
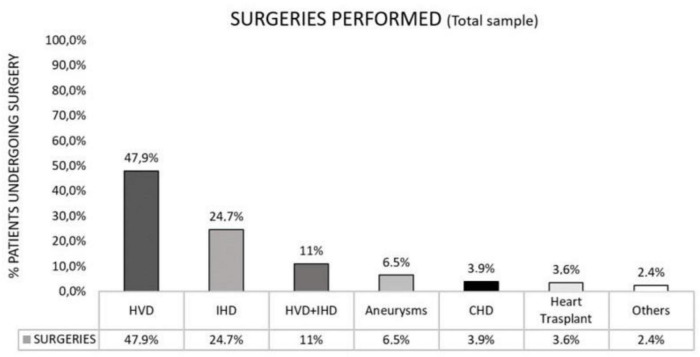
Surgeries performed among the study population.

In relation to previous medical history of non-CVD, 18.3% (*N* = 61) had a history of respiratory diseases (e.g., SAS, COPD, or others), 19.5% (*N* = 65) neurological diseases (previous stroke or others), 17.8% (*N* = 59) suffered from musculoskeletal pathology (e.g., joint prostheses, low back pain, cervical pain), 7.2% (*N* = 24) had a history of psychiatric illness (e.g., depression, anxiety, psychosis) and 68% (*N* = 227) had other comorbidities not included in the previous categories (Table 1). Regarding the surgeries performed, 47.9% (*N* = 161) of the patients were operated for heart valve disease (HVD), 24.7% (*N* = 83) for isolated ischemic heart disease, 11% (*N* = 37) underwent combined surgery (heart valve and coronary surgery), 6.5% (*N* = 22) for aneurysm repair, 3.9% (*N* = 13) for congenital heart disease and 3.6% (*N* = 12) for heart transplant transplantation (Figure 2).

**FIGURE 3:**
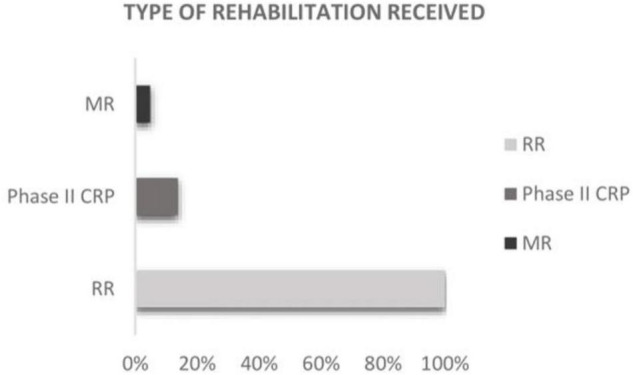
Type of rehabilitation received among the study population.

All patients were holistically evaluated by the medical rehabilitation team and received respiratory rehabilitation during their hospitalization (Figure 3). The average length of stay at the hospital was 24 days (SD ± 25.9) and 53.3% (*N* = 180) of the patients suffered some complications during the immediate post-operative period (Table 1).

Of the total of patients referred to CR (*N* = 46), most of them started the program [(90% *N* = 41)] and the average time from the referral to the beginning of phase II CR was 136 days (SD ± 98.03). Regarding the profile of patients referred to the phase II CR, there was a clear prevalence of men [71.7% (*N* = 33)] compared to women, although this did not reach statistical significance (*p* = 0.229) (Tables 2, 3). Those with more complications and longer admission received motor rehabilitation [4.8% (*N* = 16)] and at discharge, only 13.8% (*N* = 46) were referred to CR (Figure 3). Of those admitted, only 3% (*N* = 10) presented absolute contraindications for referral to CPR. Most patients were referred by the Cardiology Department: 10.9% (*N* = 5) were referred by the cardiologist at the time of hospital discharge and 73.9% (*N* = 34) were referred by the cardiologist from external consultations area in the first control after hospital discharge. The rest of the patients were referred to the program by the rehabilitation department; 6.52% (*N* = 3) were referred at the time of hospital discharge and 8.7% (*N* = 4) were derived from external rehabilitation consultations in the first control after hospital discharge.

**TABLE 2:** Characteristics of patients referred to the phase II CRP.

Variable	
Sex female (%)	28.3 (*N* = 13)
Age (%)	Average 60.8
<65	69.5 (*N* = 32)
≥65	30.4 (*N* = 14)
Healthcare area (% of patients)	–
Our hospital	43.48 (*N* = 20)
Hospital from the area	45.65 (*N* = 21)
Hospital from a different area	10.87 (*N* = 5)
CVRF (%)	89.13 (*N* = 41)
HTA	63
DL	67.4
DM	37
Overweight/obesity	19.56
Tobacco	47.8
Alcohol	2.17
Other drugs	0
Previous CV comorbidities (%)	58.7 (*N* = 27)
HF	8.7
CHD	4.3
IHD	30.4
Myocardiopathy	6.5
Pacemarker/ICD	10.9
HVD	28.3
Arrhythmia	17.4
Other comorbidities (%)	76.1 (*N* = 35)
Pulmonary disease	17.4
Neurological disease	17.4
Musculoskeletal disease	15.2
Psychiatric disease	8.7
Other disease	65.2
Diagnosis to surgery (%)	–
IHD	50 (*N* = 23)
HVD	15.2 (*N* = 7)
IHD + HVD	13 (*N* = 6)
HT	15.21 (*N* = 7)
Aneurysms	2.1 (*N* = 1)
CHD	4.3 (*N* = 2)
Others	0
Complications after surgery (%)	65.21 (*N* = 30)
Performed RR (%)	100 (*N* = 46)
Performed MR (%)	0
Hospital length stay (days)	Average 28.09 (min 8, max 131)
CVRF (%)	–
0 CVRF	8.7
1 CVRF	19.6
2 CVRF	28.3
3 CVRF	21.7
4 CVRF	19.6
5 CVRF	2.1

*N = 46 (13.8%).*

*CRP, cardiac rehabilitation program; CVRF, cardiovascular risk factors; HTA, hypertension; DL, dyslipidemia; DM, diabetes mellitus; CV, cardiovascular; HF, heart failure; CHD, congenital heart disease; IHD, ischemic heart disease; ICD, implantable cardioverter defibrillator; HVD, heart valve disease; HT, heart transplant; RR, respiratory rehabilitation; MR, motor rehabilitation; CRP, cardiac rehabilitation program.*

**TABLE 3:** Predictors of phase II cardiac rehabilitation program following cardiac surgery.

	Univariate			Age and sex adjusted			Multivariate		
			
Variable	OR	95% CI	*p*-Value	OR	95% CI	*p*-Value	OR	95% CI	*p*-Value
Sex (female)	0.67	0.3424–1.2933	0.2296	0.73	0.3702–1.4257	0.2529	1.01	0.5077–2.1754	0.2251
Age >65	0.4	0.2116–0.7619	0.0052	0.41	0.185–0.4325	<0.0001	0.32	0.1561–0.6517	0.0055
IHD surgery	3.81	2.0253–7.1752	<1e−04	4.76	2.4182–9.3669	<0.0001	3.57	1.7242–7.566	0.001
HVD surgery	0.25	0.1304–0.4819	<1e−04	0.28	0.1431–0.5406	0.0002	0.46	0.2175–0.9372	0.0241
Overweight/obesity	2.67	1.19–5.9758	0.0172	2.65	1.1591–6.0771	0.0209	2.52	1.0144–5.9889	0.0467
HT surgery	6.51	2.0084–21.1118	0.0018	4.56	1.3579–15.281	0.041			
Aneurysms surgery	0.92	0.2617–3.2339	0.8964	0.8	0.2236–2.8805	0.736			
CHD surgery	1.07	0.2293–4.9707	0.9335	0.83	0.1709–4.053	0.8202			
Other surgery	0.33	0.0757–1.4059	0.1329	0.28	0.0649–1.2346	0.093			
CVRF									
HTA	0.83	0.4437–1.5685	0.5736	1.04	0.5318–2.0313	0.9101			
DL	1.87	0.9778–3.593	0.0585	2.4	1.2165–4.7486	0.0116			
DM	1.51	0.8015–2.8602	0.2012	1.85	0.9537–3.6076	0.0687			
Tabaco	1.64	0.8879–3.0165	0.1144	1.39	0.7428–2.6111	0.3017			
>1 CVRF	0.98	0.4857–1.9861	0.9599						
>2 CVRF	1.04	0.5134–2.1058	0.9138						
>3 CVRF	2.25	1.0757–4.7155	0.0313						
Previous CV comorbidities	0.58	0.3063–1.1049	0.0979						
HF	0.66	0.2804–1.5386	0.3332						
CHD	3.01	0.5363–16.9011	0.2105						
IHD	1.68	0.8556–3.2854	0.1322						
Myocardiopathy	1.3	0.3579–4.6876	0.6934						
Pacemarker/ICD	2.44	0.8289–7.1931	0.1053						
HVD	0.52	0.2721–0.9855	0.045	0.54	0.2834–1.0456	0.0678			
Arrhythmia	0.44	0.1991–0.9846	0.0457	0.52	0.2297–1.1791	0.1176			
Other comorbidities	0.91	0.4394–1.8926	0.8045						
Pulmonary disease	0.88	0.389–1.9875	0.7571	0.95	0.4138–2.1846	0.9054			
Neurological disease	0.94	0.432–2.0616	0.8844	1	0.452–2.2209	0.9962			
Musculoskeletal disease	1.08	0.4919–2.3713	0.8479	1.19	0.5319–2.6589	0.6729			
Psychiatric disease	1.21	0.3945–3.7053	0.7397	1.18	0.3756–3.6759	0.7816			
Others	0.84	0.4406–1.5912	0.5878	0.99	0.5103–1.92	0.9758			

*IHD, ischemic heart disease; HVD, heart valve disease; HT, heart transplant; CHD, congenital heart disease; CVRF, cardiovascular risk factors; HTA, hypertension; DL, dyslipidemia; DM, diabetes mellitus; CV, cardiovascular; HF, heart failure; CHD, congenital heart disease; IHD, ischemic heart disease; ICD, implantable cardioverter defibrillator; HVD, heart valve disease.*

**FIGURE 4:**
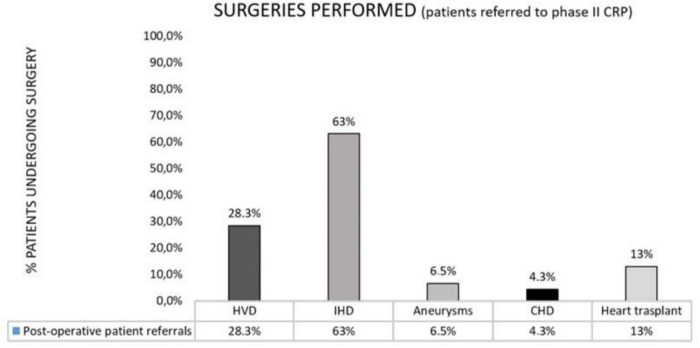
Surgeries performed among those patients referred to CR phase II.

A higher referral rate was observed in patients <65 years of age (69.56% *N* = 32) (*p* = 0.001). Regarding the reason for surgery, 50% (*N* = 23) underwent coronary surgery, 15.2% (*N* = 7) underwent heart valve surgery, 13% (*N* = 6) underwent combined surgery (heart valve and coronary surgery), 15.2% (*N* = 7) received a heart transplant, 2.1% (*N* = 1) underwent aneurysms repair surgery and only two patients (4.3%) underwent surgery for some type of congenital heart disease (Figure 4). Patients who underwent coronary surgery and heart transplantation had a greater CR referral rate (*p* = 0.001) than other surgical subpopulations (Tables 2, 3). In addition, the analysis showed that the patients with more than three cardiovascular risk factors was associated with a higher referral to CR (*p* = 0.0313) and within these, being overweight was a predictor of increased referral (*p* = 0.0172). The average hospital stay among the referred patients was 28.09 days (SD ± 26.20) and 65.21% of them (*N* = 30) suffered some complication in the immediate postoperative period. Both parameters were not significant for the referral to phase II CR.

Multivariate analysis identified age (<65 years), ischemic heart disease diagnoses, and overweight/obesity as significant independent predictors of CR referral. We did not observe an association or interaction with sex, suggesting no differences in our sample (Table 3).

## Discussion

Early rehabilitation after surgery, beginning in the intensive care unit, reduces complications in the postoperative period. The incidence of cardiopulmonary, cognitive and neuro-orthopedic complications is lower with the early onset of rehabilitation (18). Specifically, the benefits of rehabilitation in patients with CVD have been widely described. It is presumed that these improvements are the result of exercise training, psychological counseling, and education in preventive strategies (e.g., medication adherence, improved cardiovascular risk factor control) (4, 5). Despite the scientific evidence to date, the levels of referral and use of these programs are low worldwide (16, 17).

In our study, we have focused on analyzing the referral to CR programs of patients undergoing cardiac surgery. We found low levels of referral from our center in 2017 (13.8%), with rates similar to other studies. For example, the Spanish Registry of CR Units (R-EUReCa), states that “The implementation of a CRP in many countries, among which is the ours, is extremely low rehabilitating less than 10% of the candidate patients” (15). The European CR Inventory Survey shows an even lower referral in our country (<3%) (19).

The patients most frequently referred were those who underwent revascularization surgery because of ischemic heart disease. These patients are the ones who benefit most frequently from CR programs. In this pathology, CR has shown clear benefits and is strongly recommended in clinical guidelines (8). Heart transplant was the second most frequent cause of surgery among patients who were referred to our CR program. In this type of surgery, CR has also demonstrated clear benefits (11). Other frequent surgeries such as heart valve replacement or aneurysm repair also benefit from post-operative CR (10).

In the present study, regarding the predictors of CR referral in surgical patients, we found that those under 65 years had a higher referral rate. This is similar to other studies and can be explained by various personal, clinical, and social factors (20). Older adults generally have a lower perception that exercise improves health, less family support, greater risk of social isolation, and also tend to present more comorbidities. In our study, no differences were found regarding referral in patients who presented complications during the postoperative period. These patients had a longer hospital stay (more days of admission) and this variable (days of admission) also showed no differences in referral to CR programs. In contrast, patients with more cardiovascular risk factors before surgery (more specifically those patients with overweight or obesity) were more likely to be referred to CR. This may be due to the prescribing physician of CR perceiving a greater sense of illness and that these patients may be the ones who could potentially benefit most from the program.

Regarding sex, the existence of an imbalance in participation in CR programs between men and women has been documented and several studies have shown differences in attitudes toward treatment depending on the patient’s sex (21–23). In our study there was a clear tendency to lower CR referral in women, however, these differences were not statistically significant. A possible explanation contributing to this is that women are more likely to live longer than men. Specifically, ischemic heart disease in women occurs at more advanced ages, with a higher rate of acute renal failure, arrhythmias, shock, complicated diabetes, heart failure, and cerebrovascular disease. For these reasons, the low CR referral rate in these patients may be related to clinical characteristics (e.g., greater comorbidity and older age) instead of sex bias (21, 22).

Our study has several clinical implications. Although CR is a safe and effective therapy for patients after cardiovascular surgery, the design of well-defined effective strategies for the implementation of CR continues to pose a challenge in clinical practice. The success of a CR program begins with the referral of candidate patients by health professionals who manage these patients in hospitalization settings (24). Our study highlights a significant gap in clinical practice with respect to this key step. Recent studies have shown that one of the most important factors that influenced the referral to CR programs was the benefit perceived by the doctor (25). These rehabilitation units are underused, in part, due to the lack of knowledge of the prescribing professional and the concern about the safety of exercise training in these patients. We must look for solutions to increase the referral and the role of health professionals is very important.

Referral of hospitalized patients at the time of the discharge is one of the most accurate predictors that patients will being enrolled in a CR programs. Other factors are demographic factors, advanced age, low socioeconomic and cultural level, and distance to the center of CR, greater burden of morbidity (COPD, AIT, and cognitive dysfunction) (26). Additionally, the creation of referral protocols, as the one that already exists in our hospital for ischemic heart diseases, in coordination with all the professionals involved and their systematic application could improve and increase referral to CR programs (27). Added to this, coordination between hospitals must also be improved (since sometimes the patients are operated in a center different to their reference hospital) and between outpatient centers (28).

For the management of a chronic disease, it is necessary for patients to take a more active role in the daily decisions about the management of their disease. For this, the association and communication between the patient and health professionals is essential. The incorporation of trained nurses in the care system to support physicians has proven to be effective in improving the management of chronic diseases. Models like this can be implemented to improve the efficacy and adherence to these programs, resulting in better control of the patient’s disease and increasing their satisfaction (29).

While the present study provides evidence of the factors that might affect the referral rate of cardiac surgical patients to CR, caution is needed when interpreting these results. This study is retrospective, and some other factors beyond the clinical characteristics of the patients could affect CR referral rate.

## Conclusion

The benefit of CR in patients undergoing cardiac surgery has been described extensively. The beginning of rehabilitation in the early postoperative period, extends this benefit. Despite this, the level of referral to CR programs remains low. The results of this study will be part of a quality improvement project, in order to create protocols and measures that help to improve and optimize the referral of candidate patients to CRPs. The future trend should be to greater referral and participation, with special emphasis on the most affected patient groups, such as women and the elderly. PRM physicians might play a key role, in a multidisciplinary team, in order to ensure the optimal management of these complex cardiac patients. Further studies with multi-centric, larger sample size and including other socio-demographic and clinical data should be conducted.

## Data Availability Statement

The raw data supporting the conclusions of this article will be made available by the authors, without undue reservation.

## Ethics Statement

The studies involving human participants were reviewed and approved by the Gregorio Maranon Ethics Committee. Written informed consent for participation was not required for this study in accordance with the national legislation and the institutional requirements.

## Author Contributions

MS and MA-R: conceptualization. LG: formal analysis and writing – original draft preparation. MS: writing – review, editing, and supervision. JM-I, JS, and FL-J: writing – review and editing. ML and MM: data curation. MA-R: supervision. All authors have read and agreed to the final version of the manuscript and gave final approval and agreed to be accountable for all aspects of the work ensuring integrity and accuracy.

## Conflict of Interest

The authors declare that the research was conducted in the absence of any commercial or financial relationships that could be construed as a potential conflict of interest.

## Publisher’s Note

All claims expressed in this article are solely those of the authors and do not necessarily represent those of their affiliated organizations, or those of the publisher, the editors and the reviewers. Any product that may be evaluated in this article, or claim that may be made by its manufacturer, is not guaranteed or endorsed by the publisher.
